# Is Nail–Canal Diameter Discordance a Risk Factor for the Excessive Sliding of Cephalomedullary Nails in Geriatric Intertrochanteric Fracture Surgery?

**DOI:** 10.3390/medicina59061035

**Published:** 2023-05-27

**Authors:** Eic Ju Lim, Ji Wan Kim, Jeuk Lee, Chul-Ho Kim

**Affiliations:** 1 Department of Orthopedic Surgery, Chungbuk National University Hospital, Chungbuk National University College of Medicine, Cheongju 28644, Republic of Korea; limeicju@gmail.com; 2 Department of Orthopedic Surgery, Asan Medical Center, University of Ulsan College of Medicine, Seoul 05505, Republic of Korea; bakpaker@hanmail.net; 3 Department of Orthopedic Surgery, Chung-Ang University Hospital, Chung-Ang University College of Medicine, Seoul 06973, Republic of Korea; jeuklee@cauhs.or.kr

**Keywords:** intertrochanteric fracture, medullary canal, implant diameter, discordance

## Abstract

*Background and Objectives:*: There were limited studies which investigated nail diameter as a predictor for cephalomedullary nail (CMN) failure in intertrochanteric fracture (ITF). We aimed to evaluate the surgical outcomes of CMN in fragility ITF following nail–canal (N–C) diameter discordance. *Materials and Methods*: From November 2010 to March 2022, we retrospectively reviewed 120 consecutive patients who underwent CMN surgeries due to fragility ITF. We included patients with acceptable reduction and a tip–apex distance ≤ 25 mm. The N–C diameter differences both in anterior–posterior (AP) and lateral-view X-rays were measured, and we compared the number of excessive sliding instances and the rate of implant failure between the N–C concordance (≤3 mm) and discordance (>3 mm) group. Simple linear regression was used to determine the strength of the relationship between the N–C difference and sliding distance. *Results*: The sliding distance showed no differences between the groups in the AP (3.6 vs. 3.3 mm, *p* = 0.75) and lateral view (3.5 vs. 3.4 mm, *p* = 0.91). For analyses in the AP view, the AP-concordance and AP-discordance groups had 14 (25%) and 14 patients (22%) with a sliding distance of >5 mm (*p* = 0.69), while treatment failure occurred in 3 (5%) and 3 (3%) patients, respectively (*p* = 0.66). For analyses in the lateral view, the lat-concordance and lat-discordance groups had 8 (27%) and 20 patients (22%) with a sliding distance of >5 mm (*p* = 0.62), while treatment failure occurred in 1 (3%) and 4 (4%) patients, respectively (*p* = 1.00). Linear regression analyses showed that the N–C difference in either views was not a significant predictor of sliding distance in both the AP (R^2^ = 0.002, *p* = 0.60) and lateral views (R^2^ = 0.007, *p* = 0.35). *Conclusions*: If appropriate fracture reduction and fixation are achieved, the N–C discordance of short CMN does not affect treatment outcomes in ITF.

## 1. Introduction

Fragility hip fractures are becoming increasingly common and result in a significant socioeconomic burden in many countries [1]. As per the Center for Disease Control and Prevention, there are over 300,000 cases of fragility hip fractures each year in the U.S [2].

Intertrochanteric fractures (ITFs), a typical type of fragility hip fracture, comprise over 50% of all hip fractures caused by a low-energy mechanism [3,4]. For an ITF, surgery is almost always recommended as the morbidity and mortality associated with nonoperative treatment have historically been high. ITF is an extracapsular fracture; therefore, osteosynthesis is commonly recommended [3].

The cephalomedullary nail (CMN) is one of the most commonly used modalities for the fixation of fragility ITF [4]. The outcomes of CMN surgery have greatly improved with the development of fixation devices and its surgical techniques [5]. However, the fixation failure of the CMN in fragility ITF remains a sticking point.

A number of previous studies have investigated the potential risk factors for the fixation failure of CMN surgeries in ITF, including various types of instruments, initial fracture patterns, poor quality of reduction, and inappropriate implant position [6,7,8]. However, few studies have investigated nail diameter as a predictor for CMN failure. With the issue of nail diameter as a potential risk factor for CMN failure, concerns exist for both small- and large-diameter nails. A too-small nail diameter poses a possible risk of nail breakage, especially on the distal part of the nail, due to mechanical weakness. Moreover, there are concerns about the nail toggle phenomenon following nail–canal (N–C) diameter discordance [9].

On the contrary, a nail with an excessively large diameter compared to that of the femoral canal could cause iatrogenic proximal femur fracture during nail insertion or the manipulation of rotation to adjust the nail anteversion following excessive torsional force.

Therefore, in this study, we aimed to evaluate the surgical outcomes of CMN in fragility ITF following N–C diameter discordance. We set the main outcome as excessive sliding of the lag screw, which is one of the most important prodromal phenomena for the fixation failure of CMN in ITF. Moreover, we also investigated fixation failure itself, including lag screw cut-out or cut-through, as secondary outcomes.

## 2. Materials and Methods

**Figure 1:**
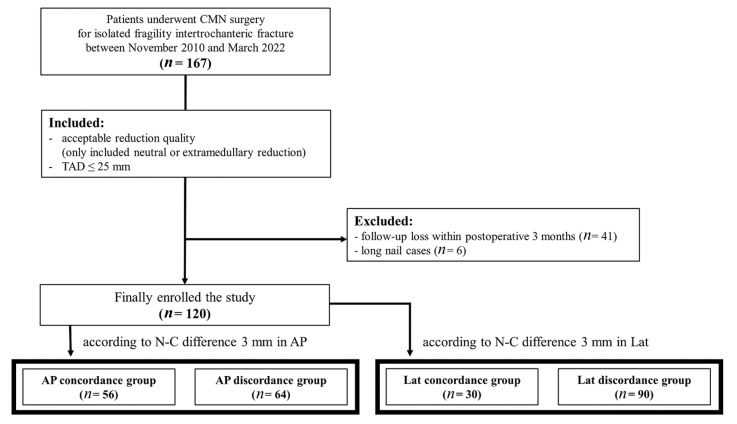
Flow diagram of patient enrollment and grouping. CMN, cephalomedullary nail; TAD, tip–apex distance; AP, anteroposterior; Lat, lateral.

This study was performed at a single institution between November 2010 and March 2022 and was approved by our institutional review board (IRB No. 2211-015-19445). We retrospectively reviewed the data of patients who underwent surgical treatment using CMN for isolated ITF injury from a low-energy mechanism of injury (falls from the standing height or less) [10]. We included (1) patients with acceptable reduction quality (neutral or extramedullary reduction) based on the description by Ito et al. [11] and (2) patients with a tip–apex distance (TAD) ≤ 25 mm [12]. Initially, 167 patients were included in this study. We excluded patients who (1) were followed up for less than 3 months postoperatively (*n* = 41) and (2) were fixed with long intramedullary nails (*n* = 6). Ultimately, 120 patients were enrolled in this study. Patients were divided into a “concordance group” or “discordance group” according to their N–C differences in the anterior–posterior (AP) and lateral views, respectively (Figure 1). The measurement of the N–C difference, sliding distance, and treatment outcome were investigated by two board-certified orthopedic surgeons with hip and pelvic trauma fellowships. Disagreements were resolved by consensus between the two investigators or by discussion with a third investigator, who was a board-certified orthopedic surgeon.

### 2.1. Measurement of N–C Difference

**Figure 2:**
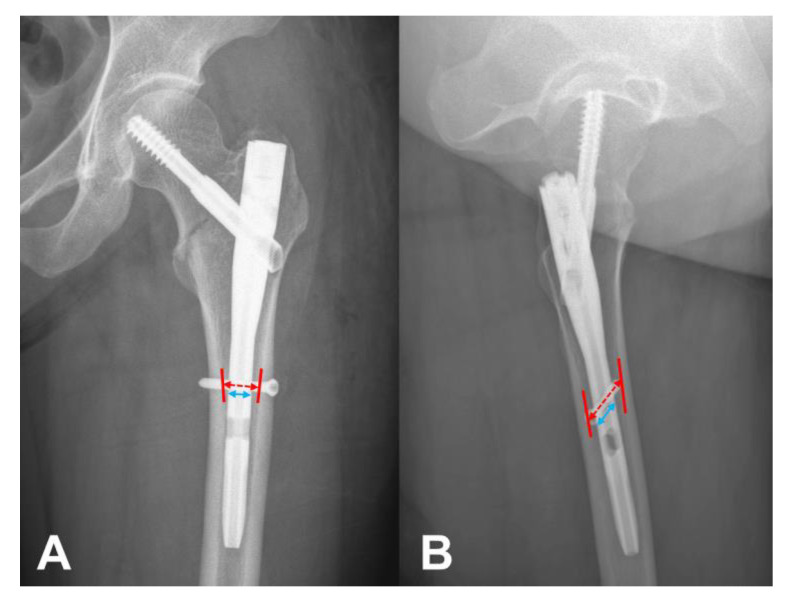
Measurement of canal and nail diameter. The canal and nail diameter were measured perpendicular to femoral anatomical axis at the level of interlocking screw both on AP (**A**) and lateral (**B**) view. The actual canal diameter was calculated through division of the measured canal diameter via magnification using real implant diameter identified in the surgical record.

We defined the N–C difference using simple radiographs, both in the AP and lateral views, with a picture archiving and communication system. The canal and nail diameters were measured perpendicular to the femoral anatomical axis at the level of the interlocking screw (Figure 2). The magnification of each radiograph was calculated using the real implant diameter identified in the surgical record. The actual canal diameter was calculated through the division of the measured canal diameter via the magnification. The N–C difference was calculated using the difference between the real canal and real implant diameter in the AP and lateral views, respectively. Patients who had an N–C difference of ≤3 mm and >3 mm were grouped into the “concordance group” and “discordance group”, respectively.

### 2.2. Data Collection and Outcome Measurement

**Figure 3:**
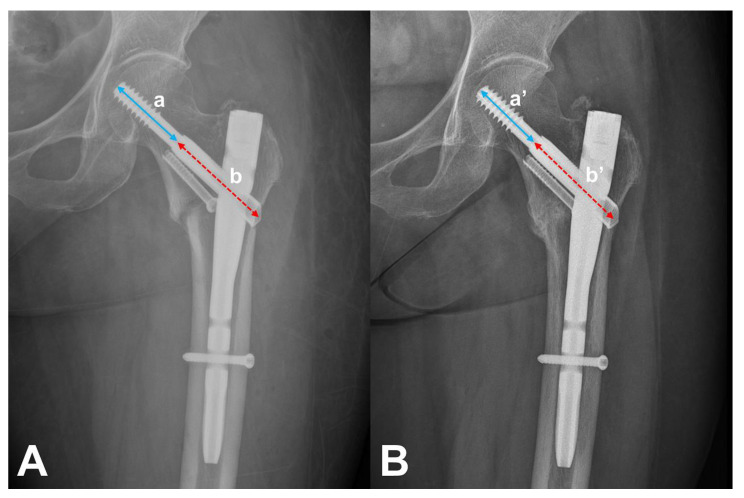
Measurement of sliding distance. The real length of each value (a, a’) was calculated using real length of barrel (b, b’) by proportional expression. Then, sliding distance was measured using immediate postoperative (**A**) and final radiographs (**B**) (a^R^ – a’^R^).

Basic demographics, including age, sex, AO/OTA classification, TAD, reduction quality, and follow-up period, were collected. Nail length, diameter, and mode of the distal locking screw (static/dynamic) were assessed using surgical records and radiographs. Sliding distance was measured using Doppelt’s method comparing immediate postoperative radiograph with the radiograph of the final follow-up or just before reoperation (Figure 3) [13,14]. Cases with a sliding distance of >5 mm were considered to have excessive sliding. The treatment outcomes were assessed as healing or treatment failure. Patients who had cortical continuity or bridging callus formation at the fracture site in at least 3 out of 4 cortical views on the simple radiographs in the final follow-up radiographs were assessed as healed patients [15]. Patients who underwent surgery due to cut-out, cut-through, or implant failure were assessed as treatment failures.

### 2.3. Statistical Analysis

The mean sliding distance, proportion of excessive sliding, and healing rate were compared between the concordance and discordance groups in both the AP and lateral views. Chi-squared or Fisher’s exact test was used for categorical variables (e.g., excessive sliding and treatment outcome), and Student’s *t*-test was used for continuous variables (e.g., mean sliding distance). Simple linear regression was used to determine the strength of the linear relationship between the N–C difference in the AP or lateral view and sliding distance. We also performed post hoc power analysis, determining whether the study was sufficiently powered to show an actual effect, even though this study was designed as retrospective in nature. Statistical significance was accepted for *p*-values of <0.05, using SPSS version 25.0 (IBM Corp., Armonk, NY, USA).

## 3. Results

The mean age at the time of surgery was 74 years (range: 40–99 years), and there were 50 males and 70 females. The most common fracture type was AO/OTA 31-A1 (51%) followed by 31-A2 (47%). Three nail lengths were used: 170 mm, 180 mm, and 200 mm. The nail diameter ranged from 9 to 13 mm, and the most common diameter was 10 mm (48%). The mean TAD was 14.6 ± 5.1 mm, and extramedullary (61%) and neutral reduction (39%) was performed (Table 1).

In the comparison of sliding distance and treatment outcome, there was no significant difference in the results between the AP and lateral views. There were no significant differences in the mean sliding distance between the concordance and discordance groups in the AP (3.6 vs. 3.3 mm, *p* = 0.75) or lateral view (3.5 vs. 3.4 mm, *p* = 0.91). For the analyses in the AP view, the concordance group had 14 patients (25%) and the discordance group had 14 patients (22%) who presented a sliding distance of >5 mm (*p* = 0.69). There were three patients (5%) with treatment failure in the concordance group and two patients (3%) in the discordance group (*p* = 0.66). For the analyses in the lateral view, the concordance group had 8 patients (27%) and the discordance group had 20 patients (22%) who presented a sliding distance of >5 mm (*p* = 0.62). There was one patient (3%) with treatment failure in the concordance group and four patients (4%) in the discordance group (*p* = 1.00) (Table 2).

**Figure 4:**
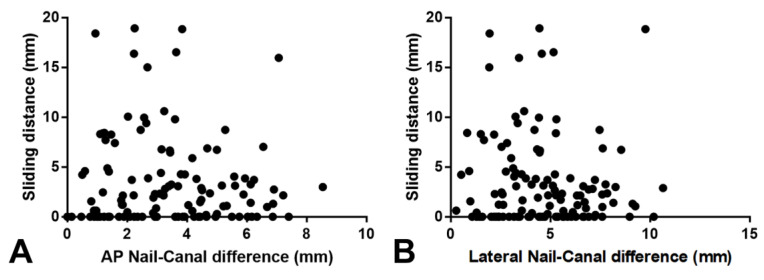
Correlations of sliding distance to nail–canal difference. Simple linear regression was used to determine the strength of the linear relationship of sliding distance to AP nail–canal difference (**A**) (R^2^ = 0.002, *p* = 0.60) and lateral nail–canal difference (**B**) (R^2^ = 0.007, *p* = 0.35) in 120 patients.

Linear regression analyses showed that the N–C difference in the AP or lateral view was not a significant predictor of excessive sliding of the lag screw (Figure 4). The N–C difference showed no or very weak association with sliding distance in both the AP (R^2^ = 0.002, *p* = 0.60) and lateral views (R^2^ = 0.007, *p* = 0.35).

We also performed a post hoc power analysis. Based on the data from the AP concordance and AP discordance groups, the post hoc power analysis showed 85.8% of power, which means there was a proper sample size and adequate power (effect size set of 0.5; alpha = 0.95; n1 = 56 (mean ± SD = 3.6 ± 5.0), n2 = 64 (mean ± SD = 3.3 ± 4.0)).

## 4. Discussion

The principal finding of the current study was that the discordance of nail diameter and femoral canal is not a risk factor for excessive sliding of the lag screw and for implant failure, including lag screw cut-out or cut-through.

Several previous studies have drawn conflicting conclusions regarding the effect of nail diameter on outcomes following intramedullary nail surgery for femoral shaft fractures. Millar et al. showed that the fit of the nail at the isthmus was directly related to the risk of exchange nailing [16]. They suggested a minimal nail fit of 70% at the isthmus (ideally, 90%). Howard et al. presented different bending stiffnesses for different nail diameters in their biomechanical study using fourth-generation composite femurs [17]. They suggested considering the appropriate nail diameter, especially for a >5 mm fracture gap. On the other hand, Serrano et al. reported, in their retrospective clinical study of 484 femoral shaft fractures, similar healing rates following different delta N–C diameters, in proximal shaft, midshaft, and distal shaft femur fractures [18]. They did not recommend the use of large-diameter nails because these nails require more canal reaming and therefore longer operative times, even with similar treatment outcomes.

However, there are very limited studies, and conflicts exist regarding the effect of nail diameter, especially in ITFs, which str more commonly encountered than femoral shaft fractures in clinical practice [19]. In a recent study, Rinehart et al. compared the rate of revision surgery due to the failure of CMN surgery between patients treated with a CMN of 10 mm and over 10 mm diameter for geriatric ITF [20]. They reported no difference in the reoperation rate between the two groups and concluded that the 10 mm nail could be chosen with comparable results to the over 10 mm diameter nail. However, they did not consider the femur geometry of each patient, which might be a potential bias. Indeed, the canal diameter varies for each individual; therefore, even nails with the same diameter could be the correct nail size for some patients and a small nail size for patients with a large canal. Moreover, Albert V. et al. reported in their more recent study of 71 patients with ITFs that a smaller N–C ratio of short CMNs was associated with increased nail toggles [9]. They concluded that increased nail toggles may be associated with increased varus collapse.

Herein, we assessed the N–C differences, not a simple nail diameter, not only in the AP view but also in the lateral view for the adjustment geometry of the femur for each patient. To the best of our knowledge, this is the first report to assess the discordance of nail to femoral canal diameter in both AP and lateral aspects, especially at the level of the distal interlocking screw hole. We believe that this method could be the most effective representation of the N–C difference compared to other previous studies.

Although the results of previous studies are controversial, the current results were comparable to Rinehart et al., which implies that we could not demonstrate the difference in treatment outcomes between the concordance and discordance of the N–C diameter [20]. We also agree that excessively large N–C discordance could be one of the risk factors for toggling of the nail, which could lead to the subsequent loss of reduction. However, this is not a problem when we obtain appropriate fracture reduction. The toggle effect can be interpreted as a change in fracture reduction, and if the appropriate fracture reduction is achieved, such as extramedullary contact of the anteromedial cortex, then a clinically meaningful toggling effect will not occur [21]. Theoretically, in the case of fracture contact on the anteromedial cortex, even if the nail is toggled laterally, reduction loss will not occur unless the fracture site is distracted. Indeed, in the current study, we only included cases that confirmed extramedullary or neutral reduction [11,21], but the previous study by Albert et al. included all consecutive cases, not only those with a good quality of reduction but also others [9]. In addition, to bear weight on the fracture site, the proximal fixation should be firm, and we only included cases with TADs of <25 mm. The results of a biomechanical study by Howard et al. also supported our results; they concluded that the larger fracture gap of femoral shaft fractures could be attributed to the reduced stiffness of the small diameter of the IM nail [17]. Thus, we believe that if we achieve a stable reduction in the fracture site during short CMN surgeries for ITF, then the nail diameter does not affect favorable treatment outcomes, because the CMN surgeries permit controlled motion by nature with appropriate reduction; this may differ in arthroplasty surgeries that require fitting of the femoral stem, which necessitates secure fixation to avoid implant loosening. Clearly, although concerns exist regarding the toggling effect, appropriate reduction and stable fixation are the most important factors to avoid treatment failure in CMN surgeries for ITF [11,21,22].

The current study had several limitations. First, there was the lack of a cause–effect relationship in the present study, which is related to the relatively small sample size. Although we demonstrated that nail canal discordance did not have a significant relationship with treatment failure, we cannot present the reason for this failure, which is due to the study’s design of using cross-sectional retrospective data. A larger sample size is needed to find the cause–effect relationship by analyzing the risk factor. Nevertheless, to the best of our knowledge, the current study included the largest number of cases among the studies that investigated the effect of nail diameter for CMN surgeries in ITFs. Second, although we evaluated the discordance between the implant and canal in both AP and lateral radiographs, only 2D images were studied. In further extended studies, 3D volumetric measurements are required. Finally, we set the primary outcome as excessive sliding of the lag screw, not implant failure or reduction failure. As we only included cases that achieved solid fracture reduction to minimize bias, there was only about 4% of treatment failure among the overall included patients; therefore, we could not directly evaluate the relationship between nail size and treatment failure. In the future, a well-structured randomized controlled analysis or meta-analysis is required to obtain more accurate results.

## 5. Conclusions

If appropriate fracture reduction and stable fixation are achieved, nail canal discordance arising from a short CMN is not an important factor in achieving satisfactory treatment outcomes in ITF.

## Figures and Tables

**Table 1:** Patient demographics, fracture patterns, implant details, surgical profiles, and follow-up durations.

Parameter	Value
Age (years)	74 ± 12 (range, 40–99)
Sex	
Male	50 (42%)
Female	70 (58%)
Fracture type (AO/OTA)	
31A1	61 (51%)
31A2	56 (47%)
31A3	3 (2%)
Nail length (mm)	
170	22 (18%)
180	81 (68%)
200	17 (14%)
Nail diameter (mm)	
9	7 (6%)
10	57 (48%)
11	18 (15%)
11.5	28 (23%)
12	3 (2%)
13	7 (6%)
Distal locking mode (n)	
Static mode	3 (2%)
Dynamic mode	117 (98%)
TAD (mm)	14.6 ± 5.1
Reduction quality *	
Extramedullary	73 (61%)
Neutral	47 (39%)
Follow-up duration (months)	16.8 (range 3–100)

TAD, tip–apex distance. * Based on the description by Ito et al. [11]

**Table 2:** Comparison between sliding distance and treatment outcome according to implant–canal differences.

	AP Concordance Group * (N–C Difference of ≤3 mm, n = 56)	AP Discordance Group * (N–C Difference of >3 mm, n = 64)	*p* Value
Mean sliding distance (mm) ^†‡^	3.6 ± 5.0	3.3 ± 4.0	0.75
Sliding distance ^§^			0.69
≤5 mm	42 (75%)	50 (78%)	
>5 mm	14 (25%)	14 (22%)	
Treatment outcome ^§¶^			0.66
Healed	53 (95%)	62 (97%)	
Treatment failure	3 (5%)	2 (3%)	
	Lateral concordance group * (N–C difference of ≤3 mm, n = 30)	Lateral discordance group * (N–C difference of >3 mm, n = 90)	*p* value
Mean sliding distance (mm) ^†‡^	3.5 ± 4.7	3.4 ± 4.4	0.91
Sliding distance ^§^			0.62
≤5 mm	22 (73%)	70 (78%)	
>5 mm	8 (27%)	20 (22%)	
Treatment outcome ^§¶^			1.00
Healed	29 (97%)	86 (96%)	
Treatment failure	1 (3%)	4 (4%)	

AP, anterior–posterior; N–C, nail–canal; statistical significance: *p*-value < 0.05. * The canal and nail diameters were measured perpendicular to the femoral anatomical axis at the level of the interlocking screw in AP and lateral view, respectively. ^†^ Mean sliding distance was expressed as mean ± standard deviation. ^‡^ Student’s *t*-test was used for comparison of mean sliding distance. ^§^ Chi-squared and Fisher’s exact test was used for comparison of sliding distance (≤5 mm and >5mm) and treatment outcome. ^¶^ Patients who had cortical continuity or bridging callus formation at the fracture site in at least 3 out of 4 cortical views on simple radiographs at final follow-up radiographs were assessed as healed patients. Patients who underwent surgery due to cut-out, cut-through, or implant failure were assessed as treatment failures.

## Data Availability

The data presented in this study are available on request from the corresponding author. The data are not publicly available due to conditions of the ethics committee of our university.
